# Enhanced Cholesterol-Lowering and Antioxidant Activities of Soymilk by Fermentation with *Lactiplantibacillus plantarum* KML06

**DOI:** 10.4014/jmb.2306.06036

**Published:** 2023-07-20

**Authors:** Ji Seung Han, Jae Yeon Joung, Hyung Wook Kim, Jin Hwan Kim, Hyo Su Choi, Hyun Jin Bae, Ji Hun Jang, Nam Su Oh

**Affiliations:** 1 Department of Food and Biotechnology, Korea University, Sejong 30019, Republic of Korea; 2 Department of Biotechnology, College of Life Sciences and Biotechnology, Korea University, Seoul 02841, Republic of Korea; 3 Department of Bio-integrated Science and Technology, College of Life Sciences, Sejong University, Seoul 05006, Republic of Korea

**Keywords:** Fermented soymilk, *Lactiplantibacillus plantarum*, isoflavone, cholesterol-lowering activity, antioxidant activity

## Abstract

This study aimed to evaluate the cholesterol-lowering and antioxidant activities of soymilk fermented with probiotic *Lactobacillaceae* strains and to investigate the production of related bioactive compounds. *Lactiplantibacillus plantarum* KML06 (KML06) was selected for the fermentation of soymilk because it has the highest antioxidant, cholesterol-lowering, and β-glucosidase activities among the 10 *Lactobacillaceae* strains isolated from kimchi. The genomic information of strain KML06 was analyzed. Moreover, soymilk fermented with KML06 was evaluated for growth kinetics, metabolism, and functional characteristics during the fermentation period. The number of viable cells, which was similar to the results of radical scavenging activities and cholesterol assimilation, as well as the amount of soy isoflavone aglycones, daidzein, and genistein, was the highest at 12 h of fermentation. These results indicate that soymilk fermented with KML06 can prevent oxidative stress and cholesterol-related problems through the production of soy isoflavone aglycones.

## Introduction

Increased dietary cholesterol intake can lead to hypercholesterolemia, defined as high blood cholesterol levels. Hypercholesterolemia is accompanied by an increase in low-density lipoprotein cholesterol and triglycerides and a decrease in high-density lipoprotein cholesterol [1]. However, soybean and soy-based products are widely used worldwide because of their abundance of bioactive compounds, such as proteins or peptides, isoflavones, saponins, and protease inhibitors [2]. Particularly, soy isoflavones, non-steroidal phytoestrogenic flavonoid molecules, have been determined the preventive effects on heart disease as well as metabolic diseases, menopause symptoms, osteoporosis, and some cancers through their hormonal and antioxidant activities [3-5]. Soy isoflavones are present in various forms such as β-glycosides, acetyl-glycosides, malonyl-glycosides, and aglycones. In particular, the bioconversion of isoflavones from glycoside forms to their aglycones, which is performed by β-glucosidase, is important for improving biological activities, as glycoside forms have lower absorption (in the small intestine), bioavailability, and bioactivity than the aglycone forms [6, 7]. Many studies have reported that lactic acid bacteria (LAB) exhibit β-glucosidase activity, and it has been suggested that the fermentation of soybeans using LAB may improve their biological activities for human health [7-9]. LAB have been used as probiotics to reduce reactive oxygen species (ROS) and oxidative stress and to improve associated diseases owing to high antioxidant activity [10-14]. It is also well known that LAB not only inhibits cholesterol synthesis by oxidative stress [15] but can also help reduce cholesterol content in the body by inhibiting cholesterol synthesis through 3-hydroxy-3-methylglutaryl-coenzyme A reductase activity and increased cholesterol excretion [16].

Therefore, this study aimed to investigate the enhancement effect of fermented soymilk with specific *Lactobacillaceae* strain on cholesterol-lowering and antioxidant activities. Subsequently, the bioactive compounds regarding soy isoflavone were identified.

## Materials and Methods

### Bacterial Strains

Previously, 10 *Lactobacillaceae* strains were isolated from Korean kimchi, and their probiotic properties were estimated in a gastrointestinal tract model, which showed great resistance to acid and bile salts and adhesion to the intestine in HT-29 intestinal cells (data not shown). These strains were identified using the V3 and V4 regions of 16S rRNA sequencing (Table S1). All *Lactobacillaceae* strains were preserved in de Man, Rogosa and Sharpe (MRS) (Difco, Detroit, USA) broth with 50% (v/v) glycerol as a cryoprotectant at -80°C. The strains were incubated aerobically at 37°C in MRS broth and subcultured thrice before conducting all experiments.

### Determination of Antioxidant Activity

The radical scavenging activities against 2-diphenyl-1-picrylhydrazyl (DPPH) and hydroxyl radicals and their ability to reduce iron ions were measured. The DPPH and hydroxyl radical scavenging activities were estimated according to the method described by Oh *et al*. [13] and Li *et al*. [17], respectively. Reducing power was examined using the ferric reducing antioxidant power (FRAP) assay [18].

### Determination of Cholesterol Assimilation

The cholesterol-lowering activity was determined using a cholesterol assimilation assay described by Choi and Chang [19]. Briefly, samples were incubated in MRS broth containing 0.5% (w/v) bovine bile (Sigma-Aldrich, USA) and 0.1 g/l cholesterol (Kanto Chemical Co., Inc., Japan). The amount of residual cholesterol was estimated by ortho-phthalaldehyde (OPA) method.

### Determination of β-Glucosidase Activity

The β-glucosidase activity was evaluated based on the hydrolysis rate of *p*-nitrophenyl β-D-glucopyranoside (p-NPG), following the method of Rekha and Vijayalakshmi [7], with a slight modification. Briefly, the samples were incubated with p-NPG, and the amount of p-nitrophenol (p-NP) released was determined at 405 nm using a spectrophotometer (Bio-Tek Instruments, USA). One unit (U) of the enzyme activity was defined as the amount of enzyme that released 1 μmol of p-NP from the substrate per milliliter per minute at 37°C under assay conditions.

### Whole Genome Sequencing and Comparative Genomic Analysis

Whole genome sequencing of the selected *Lactobacillaceae* strain, *Lactiplantibacillus plantarum* KML06, was performed de novo using a Pacific Biosciences RSII sequencer (PacBio, USA). A 20 kb library was prepared using the PacBio DNA Template Prep Kit 1.0 (Pacific Biosciences, USA). The SMRTbell™ templates were annealed using a PacBio DNA/Polymerase Binding Kit P6. Sequencing analysis was performed by Macrogen, Inc. (Korea). The resulting contigs were scaffolded using Illumina Hiseq-X (Illumina, USA). For comparative genomic analysis, the genomes of four reference strains of other *Lactobacillaceae* strains (*L. plantarum* BLS41 (GCF_002116955.1), *L. plantarum* LP3 (GCF_002286275.1), *L. plantarum* B21 (GCF_000931425.2), and *L. plantarum* pc-26 (GCF_006770485.1)) were obtained from the NCBI database (https://www.ncbi.nlm.nih.gov/genome/) and were used to carry out comparative genomic analysis using pan-genome analysis. Roary v1.007001 was used for pan-genome analysis. A phylogenetic tree was constructed using FastTreeMP v2.1.11, using the core gene alignment results generated by Roary. Dendrograms and Venn diagrams were constructed based on gene content (presence or absence) and pan-genome orthologous groups (POGs), respectively.

### Fermentation of Soymilk with Selected *Lactobacillaceae* Strain

Soymilk was prepared from a mixture of soybean [*Glycine max* (L.) Merr.] and water in a ratio of 1:3 w/w (500 g of soybeans in 1,500 g of water). The mixture was boiled for 10 min and ground for 15 min. Following grinding, the resultant slurry was filtered through a 130 mesh nylon filter cloth and sterilized at 90°C for 15 min. The sterilized soymilk was cooled to 40°C and inoculated with the selected strain of *L. plantarum* KML06, which was sub-cultured three times, washed twice, and resuspended in saline (0.85% NaCl), approximately 7 log CFU/ml. Fermentation of the mixture was performed at 37°C for 48 h. Fermented soymilk was stored in a deep freezer at -80°C until use. The chemical compositions of non-fermented and fermented soymilk samples are listed in Table S2.

### Determination of Viable Cell Counts, pH, and Degree of Hydrolysis

Changes in viable cell counts, pH, and degree of hydrolysis during 48 h of fermentation were determined at 6 h intervals. The proteolytic activity of fermented soymilk was tested using OPA, according to the method described by Church *et al*. [20]. The OPA reagent was prepared by mixing 25 ml of 0.1 M sodium tetraborate, 2.5 ml of 20%(w/w) sodium dodecyl sulfate, 1 ml of OPA (40 mg OPA/ml of methanol), and 100 μl of β-mercaptoethanol and adding distilled water to a final volume of 50 ml. Fermented soymilk was centrifuged (7,800 ×*g*, 10 min, 4°C) and 180 μl of the OPA reagent was added to 10 μl of the supernatant. The mixture was reacted for 2 min at room temperature and the absorbance was measured at 340 nm using a spectrophotometer.

### Isoflavone Analysis

**Identification of isoflavone using HPLC-ESI-MS/MS.** HPLC-ESI-MS/MS analysis was performed using 2695 HPLC (Waters Corp., USA) coupled with a Micromass Quattro Micro API benchtop triple quadrupole mass spectrometer (Waters Corp.). The analysis was carried out using ESI in the positive ion mode, and Masslynx™ software v4.1 was used to control the instrument and acquire the data. A Capcell Pak C18 reversed-phase column (250 mm × 4.6 mm id, 5 μm, Shiseido, Japan) was used to separate the isoflavones. The column temperature was maintained at 25°C, and the injection volume of the standard and sample was 20 μl. Gradient systems with 0.1%(v/v) acetic acid in distilled water (solvent A) and 0.1% (v/v) acetic acid in ACN (solvent B) were used. The flow rate of the mobile phase was 0.8 ml/min, and the mobile phase for the HPLC was as following:0–2.5 min 80% A, 2.5–10 min 70% A, 10–20 min 65% A, 20–25 min 60% A, 25–30 min 60% A, 30–32 min 80% A, and 32–42 min 80% A. The source and desolvation temperatures were set to 110 and 250°C, respectively. Argon was used as the collision gas.

**Quantitative analysis of isoflavone.** Six isoflavones (daidzin, genistin, glycitin, daidzein, genistein, and glycitein) were extracted according to the method described by Zhang and Schwartz [21]. Isoflavone β-glycosides and aglycones of non-fermented and fermented soymilk were quantified by 2695 HPLC (Waters Corp.) equipped with a C18 reversed-phase column (250 mm × 4.6 mm id, 5 μm, Shiseido, Japan) and PDA (Waters Corp.), according to the method of Marazza *et al*. [22] with some modification. Briefly, the mobile phase was 80% A (20%B), maintained for 2.5 min and decreased to 70% A for 7.5 min, and 65% A for 10 min. Subsequently, it was reduced to 60% A for 5 min, held for 5 min, increased to 80% A for 2 min, maintained for 10 min, and the analysis was terminated. The analysis was conducted for 42 min at 250 nm for daidzin and daidzein, or at 260 nm for genistin, genistein, glycitin, and glycitein.

### Statistical Analysis

All experimental data are presented as the mean ± standard deviation (SD) of triplicate measurements. SPSS software (version 25.0; IBM, USA) was used for statistical analysis, and a one-way analysis of variance (ANOVA) followed by Tukey’s multiple comparison test was used to evaluate the statistical significance between the groups. Statistical significance was set at *p* < 0.05.

## Results

### Antioxidant, Cholesterol Reduction, and β-Glucosidase Activities of the *Lactobacillaceae* Strains

**Fig. 1:**
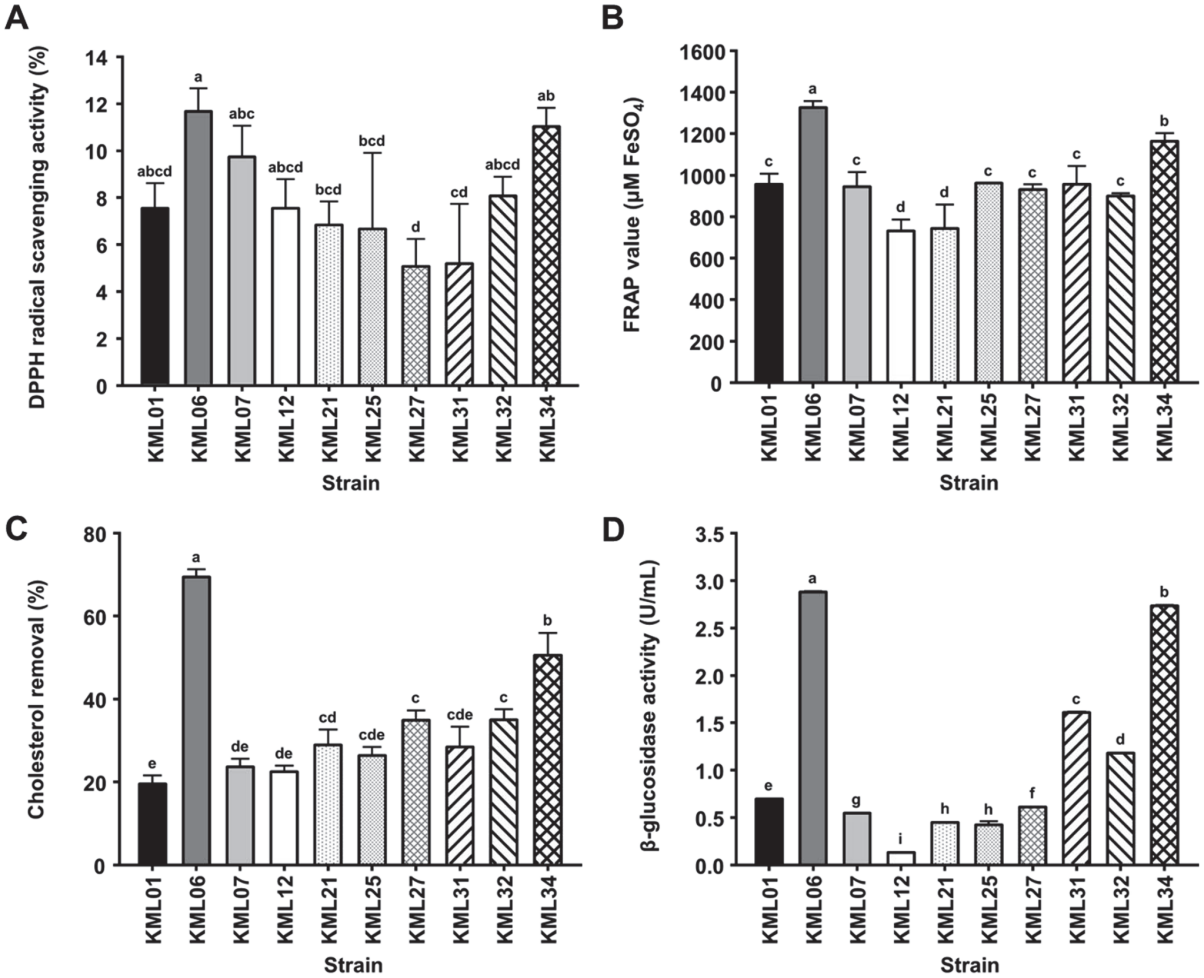
Antioxidant, cholesterol-lowering, and β-glucosidase activities of selected *Lactobacillaceae* strains. (**A**) DPPH radical scavenging activity, (**B**) Ferric reducing antioxidant power, (**C**) cholesterol assimilation, (**D**) β-glucosidase activity. Values are represented as the mean ± SD. Error bars represent the standard deviation (*n* = 3). Different small letters represent statistically significant differences (*p* < 0.05).

The antioxidant activities of the ten selected *Lactobacillaceae* strains were estimated by determining their reducing power and radical scavenging activity (Fig. 1A and 1B). The DPPH radical scavenging activity and reducing power of KML06 were significantly higher than those of the other strains. Moreover, the cholesterol reduction and β-glucosidase activities of KML06 were the highest among the strains with values of 69.5% and 2.88 U/ml, respectively (Fig. 1C and 1D). As strain KML06 showed the highest values in all assays, KML06 was selected for the fermentation of soymilk.

### Genomic Property of *Lactiplantibacillus plantarum* KML06

**Fig. 2:**
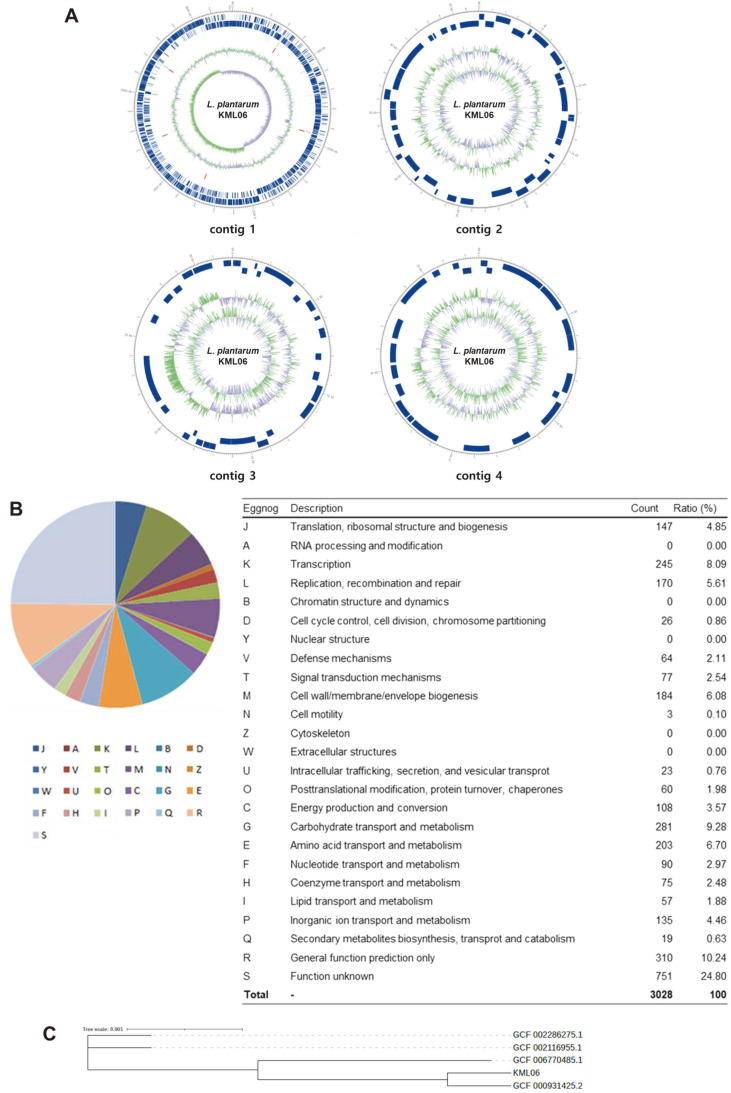
Whole genome sequencing and comparative genomics of *L. plantarum* KML06. (**A**) Circular map of *L. plantarum* KML06 genome. The circular map was drawn by applying the annotation result. Marked characteristics are shown from outside to the center; coding DNA sequence (CDS) on the forward strand, CDS on the reverse strand, tRNA (light green), rRNA (red), GC content (Region that has higher GC percentage than average is denoted in the exterior light green peak, while the other region is described in the interior lavender peak. The height of the peak describes the difference from the average GC percentage.), and GC skew (According to the formula, (G-C)/(G+C), the positive value shows that G is dominant, while the negative value shows that C is dominant. The exterior light green peak describes the region that has higher G content, while the interior lavender peak describes the region that has higher C content.). (**B**) Distribution of the functional annotation results using Clusters of Orthologous Groups (COGs) categories. (**C**) Phylogenetic tree constructed using FastTreeMP v2.1.11 using core gene alignment results generated by Roary.

Whole genome sequencing and comparative genomic analyses of KML06 were performed to confirm the functionality and novelty of this strain, respectively. General genomic information of KML06 is shown in Table 1 and the circular contig is shown in Fig. 2A. A total of 72,105 reads with an average length of 10,343 bp (745,787,272 total subread bases) were obtained, and the genome contained 3,319,595 bp with a G + C content of 44.44%. Moreover, the KML06 genome consisted of four contigs with N50 values of 3,213,056 bp. The genome of KML06 was composed of 3,077 coding DNA sequences (CDSs), 16 rRNA genes, and 69 tRNA genes. The distribution of the Clusters of Orthologous Group (COG) categories is shown in Fig. 2B. The most common COG category was S (unknown function), followed by R (general function prediction only), G (carbohydrate transport and metabolism), K (transcription), E (amino acid transport and metabolism), and M (cell wall/membrane/envelope biogenesis). In addition, the genome of KML06 was compared with those of four different *L. plantarum* strains pan-genome analysis (Fig. 2C). The phylogenetic tree constructed based on the ortho-ANI value indicated that KML06 was a novel genomic strain, although it is located close to the reference strains.

### Growth Kinetics and Changes in pH and Lactic Acid Content

**Fig. 3:**
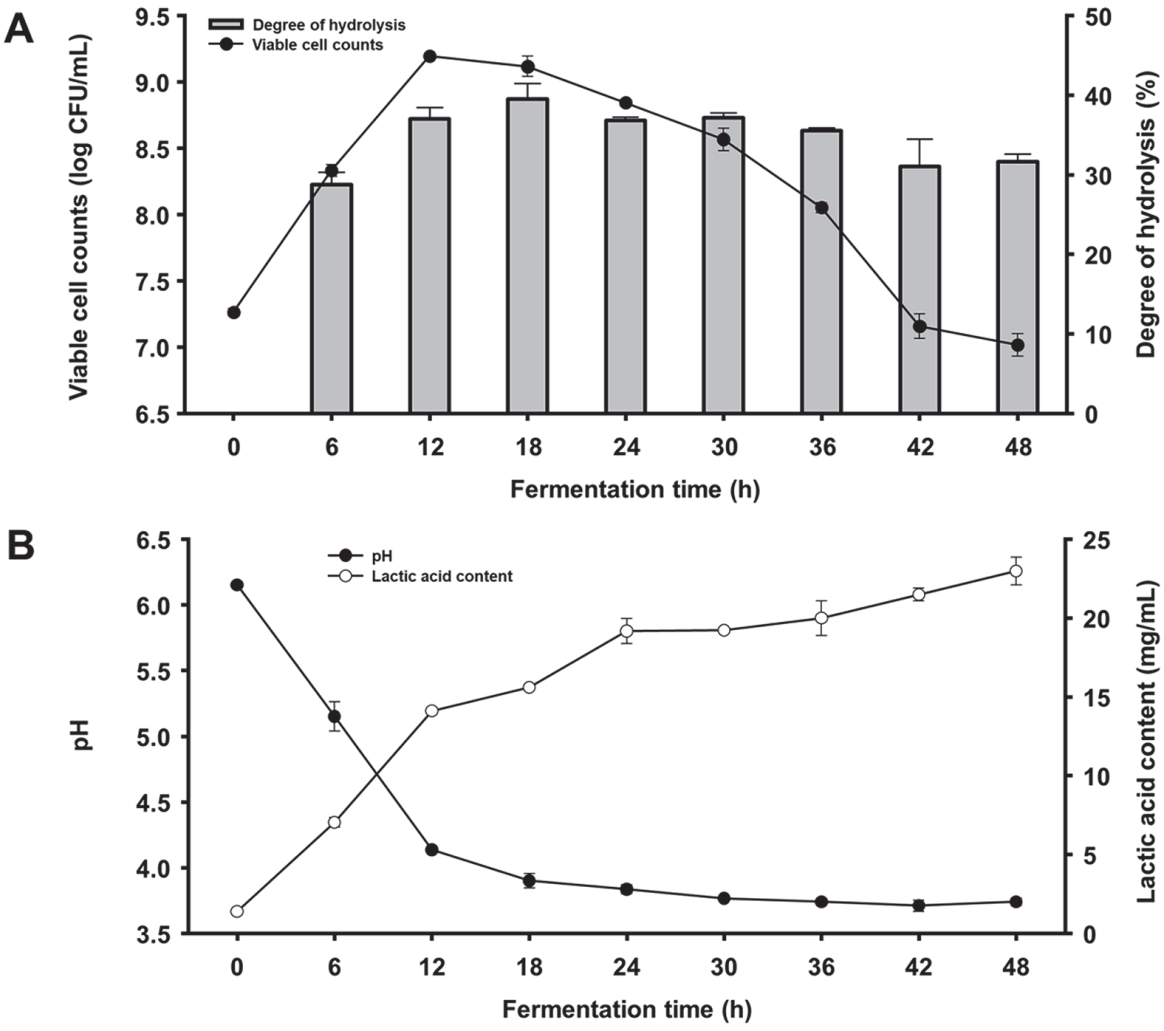
Changes in (A) viable cell counts and degree of hydrolysis and (B) pH and lactic acid content during soymilk fermented with *L. plantarum* KML06. Values are represented as the mean ± SD. Error bars represent the standard deviation (*n* = 3).

To evaluate changes in microbiological properties during soymilk fermentation with KML06, the viable cell count, degree of hydrolysis, pH, and lactic acid content were measured (Fig. 3). The number of KML06 cells exponentially increased immediately after soymilk inoculation. The viable cell count increased from 7.26 log CFU/ml to 9.20 log CFU/ml until 12 h of fermentation but decreased slowly until the end of the fermentation, reaching a level similar to that at the beginning of fermentation. Soy proteins were degraded by KML06 and the number of liberated peptides increased during the exponential phase. Similar to the growth of KML06, lactic acid content increased rapidly during the exponential phase and showed a relatively small change after 24 h of fermentation. However, the pH rapidly decreased from 6.15 to 3.90 after 18 hours of fermentation.

### Changes in Antioxidant and Cholesterol Reduction Activities of Fermented Soymilk during Fermentation

**Fig. 4:**
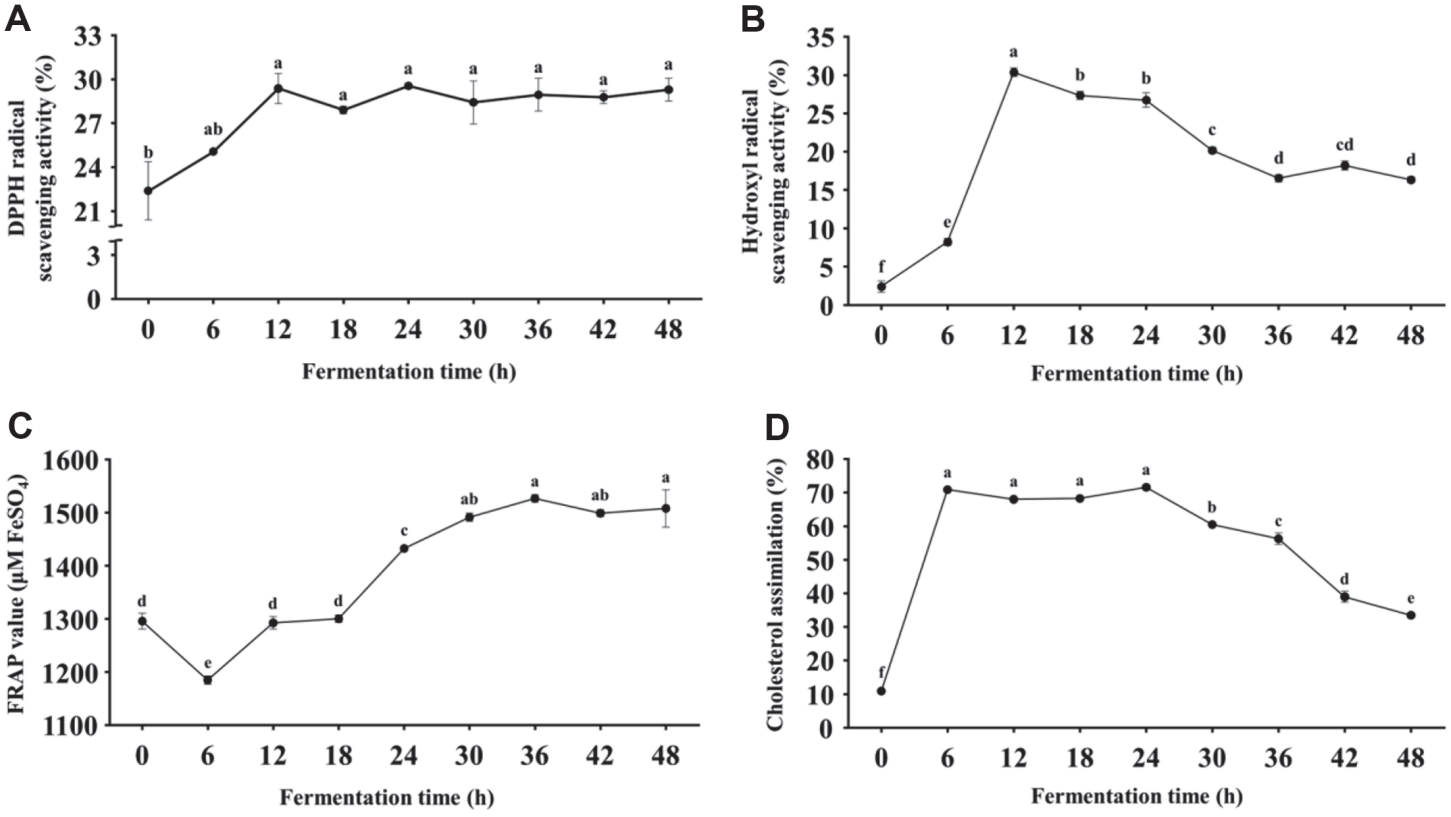
Antioxidant and cholesterol reduction activities of soymilk fermented with *L. plantarum* KML06 during the fermentation period. (**A**) DPPH radical scavenging activity, (**B**) hydroxyl radical scavenging activity, (**C**) ferric reducing antioxidant power, (**D**) cholesterol assimilation. Error bars represent the standard deviation (*n* = 3). Different small letters represent statistically significant differences (*p* < 0.05).

Changes in the antioxidant and cholesterol reduction activities of fermented soymilk were measured during fermentation (Fig. 4). DPPH and hydroxyl radical scavenging activities and reducing power increased by fermentation of soymilk with KML06. In particular, the hydroxyl radical scavenging activity increased exponentially upto 12 h of fermentation but showed a decreasing trend until the end of fermentation, whereas the DPPH radical scavenging activity and reducing power steadily increased until the end of fermentation. In addition, the cholesterol-reducing ability rapidly increased during 6 h of fermentation, but there was no change until 24 h of fermentation and then decreased until the end of fermentation as a result of hydroxyl radical scavenging activity.

### Identification and Quantification of Isoflavones

**Fig. 5:**
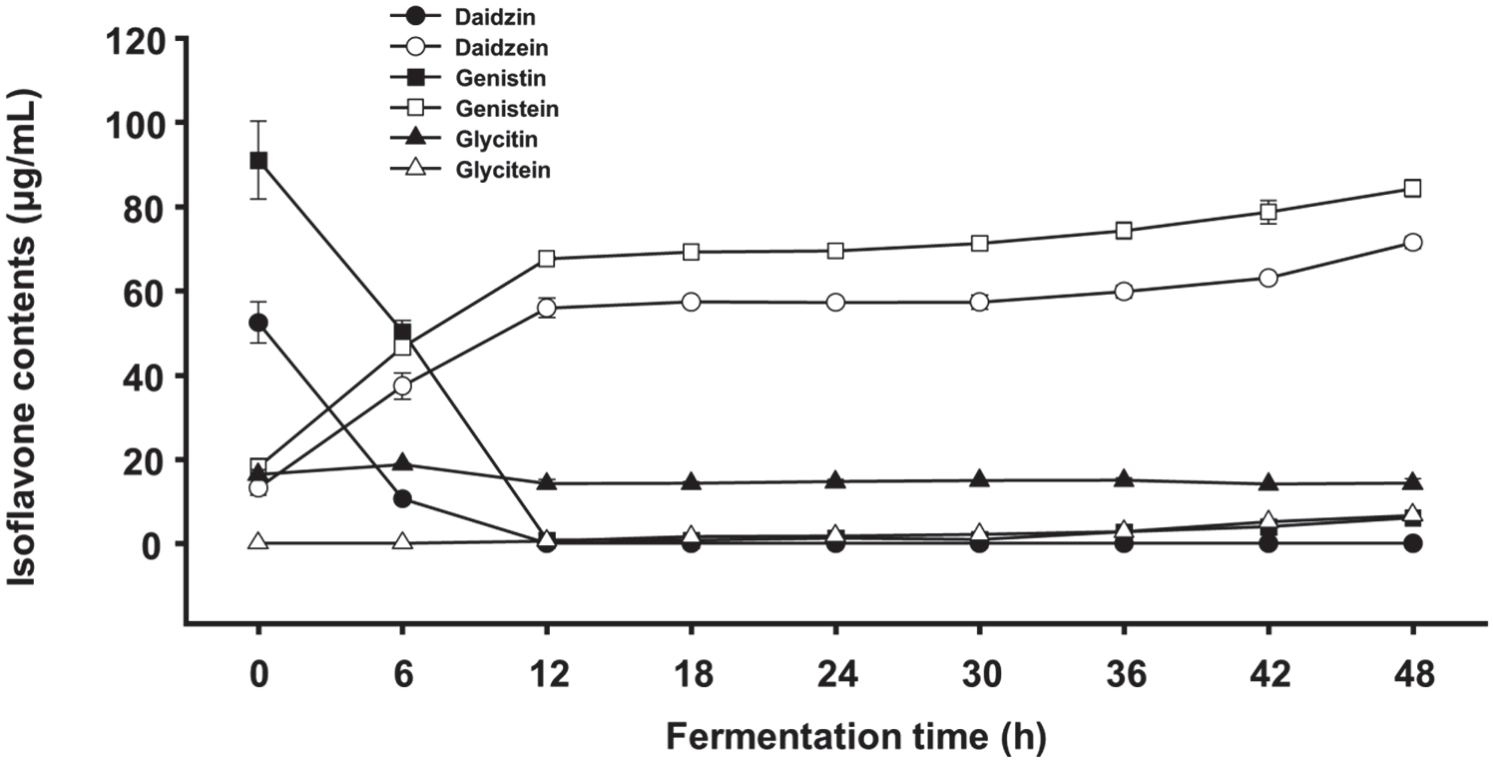
Changes in the isoflavone content of soymilk fermented with *L. plantarum* KML06 during the fermentation period. Error bars represent the standard deviation (*n* = 3). Different small letters within a column represent statistically significant differences (*p* < 0.05).

**Fig. 6:**
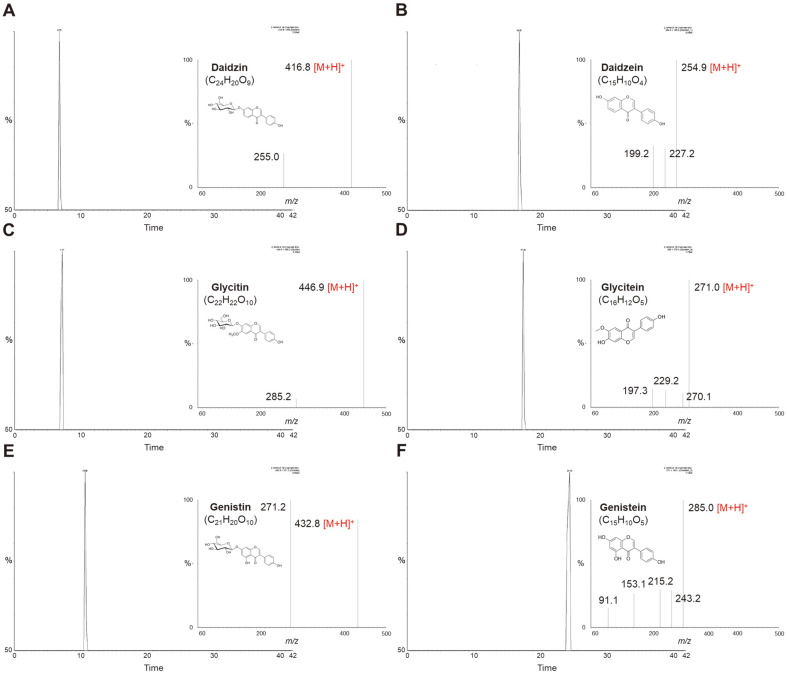
Mass fragmentation patterns of identified isoflavones. (**A**) Daidzin, (**B**) daidzein, (**C**) glycitin, (**D**) glycitein, (**E**) genistin, and (**F**) genistein.

Changes in the amounts of the six isoflavone glycosides and aglycones in soymilk during fermentation by KML06 are shown in Fig. 5 and Table 3. Before soymilk fermentation, the amounts of isoflavone β-glycosides, such as genistin, daidzin, and glycitin, were higher than those of their aglycones, including genistein, daidzein, and glycitein. Among the β-glycosides, the amount of genistin (91.1 μg/ml) was the highest, followed by that of daidzin (52.5 μg/ml). During the first 12 h of fermentation, the amounts of genistin and daidzin rapidly decreased. In contrast, genistein and daidzein levels increased until 12 h of fermentation. Daidzin was not detected from 12 h until the end of fermentation. Additionally, the amount of glycitin steadily decreased until the end of fermentation. In addition, six isoflavones were identified using HPLC-MS/MS. Table 2 shows the retention times, mass spectral characteristics, and multiple reaction monitoring transitions for each isoflavone. The mass spectra of the isoflavones are shown in Fig. 6.

## Discussion

Soy isoflavones are important because of their biological activities in the prevention of heart diseases, metabolic diseases, menopause symptoms, osteoporosis, and certain cancers [3-5]. They predominantly exist as glucosides, -glycosides, acetyl-glycosides, malonyl-glycosides, and aglycones [8]. In particular, fermentation enhances the presence of aglycone forms in soy products through the action of bacterial β-glucosidases, which are recognized as a crucial mechanism leading to higher bioavailability of the aglycone forms compared to the glucoside forms [6, 7]. Lim *et al*. [23] found that daidzein and genistein, the main soy isoflavone aglycones, were produced through soybean fermentation. They also observed that these compounds decreased serum total cholesterol levels and increased high-density lipoprotein cholesterol levels in mice fed a high-cholesterol diet. In addition, dietary soy isoflavones have been reported to have regulatory functions in cholesterol and fatty acid metabolism, and these functions are more effective in isoflavone aglycones than in isoflavone glucosides [24].

In this study, we determined the cholesterol-lowering and antioxidant activities of soymilk fermented with a specific probiotic *Lactobacillaceae* strain. The probiotic strain *L. plantarum* KML06 was selected based on its highest antioxidant, cholesterol-lowering, and β-glucosidase activities among the candidate strains isolated from Korean kimchi. Several studies have shown that *Lactobacillaceae* species as probiotics have protective effects against various diseases through their health benefits [25], including lowering ROS and oxidative stress, thus alleviating oxidative stress-related diseases through high antioxidant activity [10-14]. It is also well known that probiotic *Lactobacillaceae* species not only inhibit cholesterol synthesis but also help reduce cholesterol content [15, 16, 26]. A previous study reported that the cell-free supernatant of *L. plantarum* inhibited cholesterol synthesis through the phosphorylation of AMP protein kinase, which reduced the expression of 3-hydroxy-3-methylglutaryl-coenzyme A reductase [27]. Moreover, supplementation of *L. plantarum* diet in patients with hypercholesterolemia significantly contributes to a reduction in serum cholesterol [28]. Additionally, soy proteins are hydrolyzed into peptides and free amino acids through fermentation with probiotic strains, resulting in improved preventive effects against various diseases, including antioxidant, antihypertensive, hypocholesterolemic, and anti-diabetic effects on the host [5, 29, 30]. In the present study, soymilk fermented using the probiotic strain KML06 exhibited significantly higher viable cell counts, degree of hydrolysis, antioxidant activities in radical scavenging and FRAP assays, and cholesterol-reducing activity than non-fermented soymilk. In particular, the values were the highest at 12 h of fermentation, and the levels of soy isoflavone aglycones daidzein and genistein, which are the two major aglycones produced by bacterial fermentation [31], were also detected to be the highest at 12 h of fermentation. These results are consistent with those of previous studies that showed that the bioconversion of glycosides to aglycones tends to increase and then decrease with increasing fermentation time [31, 32]. This could be due to the β-glucosidase produced by KML06, as the viable cell count of KML06 was the highest at 12 h of fermentation. During fermentation, the bioconversion of isoflavones from their glycoside forms to their aglycones is performed by microbial enzymes, including β-glucosidase, which hydrolyzes β-1,4 glycosidic bonds. However, since these enzymes do not exist in humans, microorganisms that represent β-glucosidase activity play an important role in the biotransformation of soy isoflavones in the human body [8]. Isoflavone aglycones are absorbed faster into the mucosa of the small intestine and have higher antioxidant activity than their glycoside forms [33, 34]. In addition, their lipolytic effects have been previously reported in rats fed a high-fat diet [31]. According to previous studies, the estrogenic activities of soy isoflavones are responsible for the lipolytic effects of soy proteins, leading to decreased serum cholesterol and triglyceride levels, thereby reducing the incidence of cardiovascular diseases [24]. The metabolites of dietary isoflavones are believed to modulate hepatic cholesterol 7a-hydroxylase mRNA levels; the mRNA levels of cytochrome p450-2A and phosphoribosyl pyrophosphate synthase-associated protein were upregulated in gerbil liver after the consumption of soy isoflavones [35]. The viable cells of the probiotic KML06 and soy isoflavone aglycones in fermented soymilk might help increase their antioxidant and cholesterol-lowering activities.

In conclusion, soymilk fermented with the probiotic strain *L. plantarum* KML06 as a new functional ingredient could increase the therapeutic potential effect through its antioxidant- and cholesterol-assimilating activities derived from isoflavone aglycones. However, it is important to note that the regulative function of soy isoflavones on lipid metabolism still needs to be determined.

## Supplemental Materials

Supplementary data for this paper are available on-line only at http://jmb.or.kr.



## Figures and Tables

**Table 1:** General genomic information of *Lactiplantibacillus plantarum* KML06.

	*L. plantarum* KML06
Sequencing platforms	Pacbio RSII
Assembler	illumina HiSeq-X
Number of subreads	72,105
Average subread length (bp)	10,343
Genome size (bp)	3,319,595
G+C content (%)	44.44
Predicted CDS	3,077
Number of contigs	4
Number of rRNA genes	16
Number of tRNA genes	69
N50 (bp)	3,213,056

**Table 2:** Selected reaction monitoring (SRM) conditions for the confirmation analysis of isoflavones via HPLC-ESI-MS/MS.

Compounds	Formula	Retention time	Target ion (*m/z*)	Product ion (*m/z*)	Cone voltage (V)	Collision energy (V)
Daidzin	C_21_H_20_O_9_	6.79	416.8	255.0	25	18
Glycitin	C_22_H_22_O_10_	7.17	446.9	285.2	20	15
Genistin	C_21_H_20_O_10_	10.66	432.8	271.2	30	18
Daidzein	C_15_H_10_O_4_	16.87	254.9	199.2	50	25
Glycitein	C_16_H_12_O_5_	17.45	285	270.1	45	25
Genistein	C_15_H_10_O_5_	24.43	271	153.1	50	25

**Table 3:** Changes in the isoflavone content of soymilk fermented with *L. plantarum* KML06 during the fermentation period.

Fermentation time (h)	Isoflavone content (μg/ml)						
	Daidzin	Daidzein	Genistin	Genistein	Glycitin	Glycitein	Total amount of aglycones
0	52.5 ± 4.9 ^a^	13.2 ± 1.7 ^e^	91.1 ± 9.3 ^a^	18.2 ± 1.9 ^f^	16.3 ± 1.2 ^b^	ND	31.5
6	10.6 ± 0.3 ^b^	37.4 ± 3.1 ^d^	50.2 ± 2.6 ^b^	46.6 ± 2.0 ^e^	18.7 ± 0.2 ^a^	ND	84.0
12	ND	55.9 ± 2.3 ^c^	0.8 ± 0.4 ^c^	67.6 ± 0.3 ^d^	14.1 ± 1.0 ^c^	0.5 ± 0.1 ^e^	124.0
18	ND	57.4 ± 0.8 ^c^	0.7 ± 0.1 ^c^	69.2 ± 0.6 ^d^	14.2 ± 0.4 ^c^	1.6 ± 0.3 ^de^	128.2
24	ND	57.2 ± 0.7 ^c^	1.2 ± 0.2 ^c^	69.5 ± 0.6 ^cd^	14.6 ± 0.6 ^bc^	1.8 ± 0.3 ^cd^	128.5
30	ND	57.3 ± 1.8 ^c^	0.9 ± 0.1 ^c^	71.3 ± 1.7 ^cd^	14.8 ± 0.1 ^bc^	2.1 ± 0.4 ^cd^	130.7
36	ND	59.8 ± 1.5 ^bc^	2.8 ± 0.2 ^c^	74.3 ± 1.9 ^bc^	14.9 ± 0.7 ^bc^	2.8 ± 0.7 ^c^	136.9
42	ND	63.1 ± 1.1 ^b^	4.0 ± 0.2 ^c^	78.7 ± 2.8 ^b^	14.0 ± 0.1 ^c^	5.2 ± 0.5 ^b^	147.0
48	ND	71.4 ± 1.5 ^a^	6.1 ± 0.5 ^c^	84.3 ± 2.1 ^a^	14.2 ± 1.1 ^c^	6.6 ± 0.3 ^a^	162.3
