# Supplementary material for: Enhanced Cholesterol-Lowering and Antioxidant Activities of Soymilk by Fermentation with Lactiplantibacillus plantarum KML06
Source: J Microbiol Biotechnol. 2023 Jul 20;33(11):1475–83. doi: 10.4014/jmb.2306.06036 (PMC10699276; doi:10.4014/jmb.2306.06036)
Supplement: Supplementary file 1 [file jmb-33-11-1475-supple.pdf]

J. Microbiol. Biotechnol.
https://doi.org/10.4014/jmb.2306.06036

# Supplementary Tables and Figure

**Supplementary Table S1. Identification of *Lactobacillus* strains used in this study**

| Strain | Species identification by 16S rRNA sequencing |
|---|---|
| KML01 | *Lactobacillus gasseri* |
| KML06 | *Lactiplantibacillus plantarum* |
| KML07 | *Lactobacillus gasseri* |
| KML12 | *Limosilactobacillus reuteri* |
| KML21 | *Limosilactobacillus reuteri* |
| KML25 | *Limosilactobacillus reuteri* |
| KML27 | *Lactobacillus gasseri* |
| KML31 | *Lactobacillus gasseri* |
| KML32 | *Lactobacillus gasseri* |
| KML34 | *Lactiplantibacillus plantarum* |

**Supplementary Table S2. Chemical composition of non-fermented and fermented soymilk.**

| | **Total solid (%)** | **Protein/TS (%)** | **Fructose (mg/mL)** | **Glucose (mg/mL)** | **Sucrose (mg/mL)** |
|---|---|---|---|---|---|
| Non-fermented soymilk | 12.95 ± 0.05 | 41.47 ± 0.31 | 2.52 ± 0.13 | 0.61 ± 0.01 | 19.76 ± 0.23 |
| Fermented soymilk (48 h) | 12.37 ± 0.10 | 43.49 ± 0.37 | 2.09 ± 0.01 | ND | ND |

**Supplementary Table S3. Changes of sugar content in fermented soymilk with *Lactiplantibacillus plantarum* KML06 during fermentation period.**

| Fermentation time (hours) | Sugar (mg/mL) | | |
|---|---|---|---|
| | Fructose | Glucose | Sucrose |
| 0 | 2.52 ± 0.13[a] | 0.61 ± 0.01 | 19.76 ± 0.23[a] |
| 6 | 2.15 ± 0.01[b] | ND | 17.85 ± 0.26[b] |
| 12 | 2.21 ± 0.07[b] | ND | 10.62 ± 0.36[c] |
| 18 | 2.10 ± 0.04[b] | ND | 7.36 ± 0.03[d] |
| 24 | 2.13 ± 0.01[b] | ND | 4.85 ± 0.08[e] |
| 30 | 2.09 ± 0.00[b] | ND | 3.60 ± 0.06[f] |
| 36 | 2.13 ± 0.01[b] | ND | 2.66 ± 0.03[g] |
| 42 | 2.11 ± 0.02[b] | ND | 1.81 ± 0.00[g] |
| 48 | 2.09 ± 0.01[b] | ND | ND |

Different letters within a row represent statistically significant differences ($p < 0.05$).

**Supplementary Table S4. Changes of organic acid content in fermented soymilk with *Lactiplantibacillus plantarum* KML06 during fermentation period.**

| Fermentation time (hours) | Organic acid content (mg/mL) | | | | |
|---|---|---|---|---|---|
| | Citric acid | Lactic acid | Formic acid | Acetic acid | Propionic acid |
| 0 | 4.77 ± 0.03[a] | 1.39 ± 0.01[f] | 0.14 ± 0.00[b] | ND | ND |
| 6 | 4.63 ± 0.14[ab] | 7.04 ± 0.22[e] | 0.12 ± 0.01[b] | 0.15 ± 0.01[e] | 8.50 ± 0.21[ab] |
| 12 | 4.27 ± 0.06[b] | 14.10 ± 0.08[d] | 0.58 ± 0.03[a] | 0.71 ± 0.02[d] | 8.77 ± 0.19[a] |
| 18 | 3.40 ± 0.01[c] | 15.60 ± 0.04[d] | 0.51 ± 0.04[a] | 0.78 ± 0.05[d] | 7.71 ± 0.04[ab] |
| 24 | 2.95 ± 0.15[cd] | 19.17 ± 0.56[c] | 0.52 ± 0.06[a] | 1.15 ± 0.01[c] | 8.41 ± 0.46[ab] |
| 30 | 2.56 ± 0.02[d] | 19.22 ± 0.10[c] | 0.45 ± 0.00[ab] | 1.16 ± 0.00[c] | 7.75 ± 0.06[ab] |
| 36 | 1.97 ± 0.08[e] | 19.98 ± 0.77[bc] | 0.36 ± 0.01[ab] | 1.24 ± 0.04[bc] | 7.28 ± 0.34[b] |
| 42 | 1.78 ± 0.02[e] | 21.47 ± 0.29[ab] | 0.37 ± 0.04[ab] | 1.41 ± 0.02[b] | 7.63 ± 0.12[ab] |
| 48 | 1.87 ± 0.07[e] | 22.97 ± 0.63[a] | 0.42 ± 0.05[ab] | 1.69 ± 0.06[a] | 8.22 ± 0.34[ab] |

Different letters within a row represent statistically significant differences ($p < 0.05$).

**Supplementary Table S5. Selected reaction monitoring (SRM) conditions for the confirmation analysis of acetyl and malonyl glucoside forms of isoflavone via HPLC-ESI-MS/MS.**

| Compounds | Formula | Retention Time | Target ion (*m*/*z*) | Product ion (*m*/*z*) | Cone voltage (V) | Collision energy (V) |
|---|---|---|---|---|---|---|
| Acetyl daidzin | $C_{22}H_{22}O_9$ | 6.29 | 458.5 | 254.8 | 40 | 20 |
| Malonyl daidzin | $C_{24}H_{22}O_{12}$ | 4.03 | 502.4 | 254.8 | 45 | 20 |
| Acetyl glycitin | $C_{24}H_{24}O_{11}$ | 6.27 | 488.5 | 284.9 | 40 | 20 |
| Malonyl glycitin | $C_{25}H_{24}O_{13}$ | 4.01 | 532.5 | 284.9 | 40 | 20 |
| Acetyl genistin | $C_{23}H_{22}O_{11}$ | 7.99 | 474.6 | 270.9 | 40 | 20 |
| Malonyl genistin | $C_{24}H_{22}O_{13}$ | 6.59 | 515.8 | 270.8 | 45 | 20 |

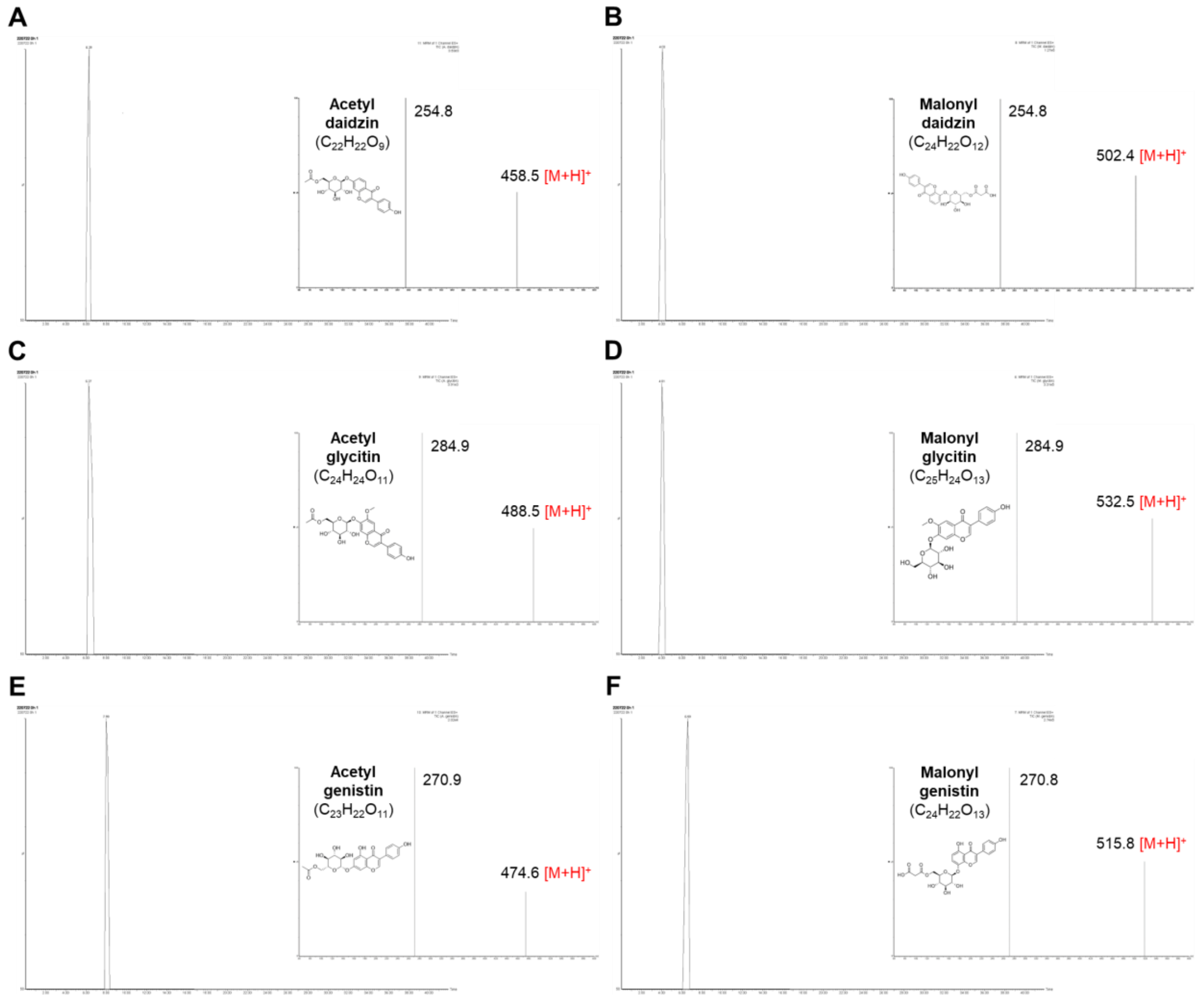


**Supplementary Figure S1. Mass fragmentation patterns of identified isoflavones.** (A) Acetyl daidzin, (B) Malonyl daidzin, (C) Acetyl glycitin, (D) Malonyl glycitin, (E) Acetyl genistin, (F) Malonyl genistin.
